# ApoptomiRs of Breast Cancer: Basics to Clinics

**DOI:** 10.3389/fgene.2016.00175

**Published:** 2016-09-29

**Authors:** Shivani Sharma, Praveen K. Patnaik, Stella Aronov, Ritu Kulshreshtha

**Affiliations:** ^1^Department of Biochemical Engineering and Biotechnology, Indian Institute of Technology DelhiNew Delhi, India; ^2^Department of Molecular Biology, Ariel UniversityAriel, Israel

**Keywords:** breast cancer, apoptosis, microRNA, apoptomiR, biomarker

## Abstract

Apoptosis, a form of programmed cell death, is a highly regulated process, the deregulation of which has been associated with the tumor initiation, progression, and metastasis in various cancers including breast cancer. Induction of apoptosis is a popular target of various therapies currently being tested or used for breast cancer treatment. Thus, identifying apoptotic mediators and regulators is imperative for molecular biologists and clinicians for benefit of patients. The regulation of apoptosis is complex and involves a tight equilibrium between the pro- and anti-apoptotic factors. Recent studies have highlighted the role of miRNAs in the control of apoptosis and their interplay with p53, the master guardian of apoptosis. Here, we summarize and integrate the data on the role of miRNAs in apoptosis in breast cancer and the clinical advantage it may offer for the prognosis or treatment of breast cancer patients.

## Introduction

Breast cancer is the most common malignancy in women in western nations with a scary current estimate of one in eight US women likely to develop invasive breast cancer once in her lifetime (American Cancer Society, 2015; Ferlay et al., 2015). The lowest incidences of breast cancer are seen in Asia and Africa but the cases are steadily rising and poor survival rates remain a cause of grave concern (American Cancer Society, 2015; Ferlay et al., 2015). The various risk factors are age, density of the breast, family and personal health history, germline mutations in the tumor suppressor genes-*BRCA1, BRCA2*, and other breast cancer susceptibility genes, alcohol intake, endogenous estrogen levels, use of hormone replacement therapy, radiation exposure to the breast, obesity, and race/ethnicity (American Cancer Society, 2015; Ferlay et al., 2015). Breast cancer is a heterogeneous disease characterized by up to 21 distinct histological subtypes and at least four different molecular subtypes each with distinct presentations, response to treatment, and clinical outcomes (American Cancer Society, 2015; Ferlay et al., 2015).

There has been marked improvement in the breast cancer survival rates and decline in deaths owing to early detection, better understanding of the disease, and personalized treatment (Ferlay et al., 2015). Current breast cancer treatment modality involves surgery (both curative and preventive), radiation therapy, systemic chemotherapy, endocrine therapy, and targeted therapy that interfere with the cellular functions thereby inducing cell cycle arrest and eventually apoptosis, a form of programmed cell death (Gonzalez-Angulo et al., 2007; Florea and Busselberg, 2013). However, breast cancer still remains the leading cause of death from cancer among women. The most significant reasons for therapy failure are tumor metastases to distant sites and development of treatment resistance (Gonzalez-Angulo et al., 2007; Florea and Busselberg, 2013). It is often seen that metastatic and treatment resistant cells lose control over apoptosis and thus fail to die in response to various therapies. Identification of the mediators and regulators of the apoptotic pathway may help to tailor treatments for maximization of treatment efficacy leading to complete tumor remission and prevention of metastases (Kasibhatla and Tseng, 2003).

Recently, microRNAs (miRNAs) have emerged as critical regulators of apoptosis. miRNAs are small non-coding RNAs (~18–25 bases) regulating gene expression at the post-transcriptional and/or translational levels. Traditionally, they are known to bind to the 3′ untranslated region of the target transcript to bring about transcript degradation or inhibition of translation depending on the extent of complementarity (Bartel, 2004). However, recent research suggests that miRNA may bind anywhere in the transcript to regulate its levels and can also bring about increase in the transcript levels by various mechanisms (Antonio et al., 2006). It is increasingly becoming clear that miRNAs generate a complex network with mRNAs, snoRNAs, long non-coding RNAs, and RNA binding proteins to establish cellular transcriptome (Mattick and Makunin, 2006).

The generation of miRNA profiles of normal or patient samples using microarray, qPCR, or deep sequencing technology and its analyses has helped in identifying useful clinical correlations of miRNAs with potential applications in cancer diagnosis, prognosis or therapy (Hammond, 2006). Several human miRNAs have been reported to be significantly deregulated in at least one cancer type and have been shown to function as oncomiRs or tumor suppressor miRs. Such deregulation of miRNA expressions across various tumor types suggests that these miRNAs may be involved in crucial cellular pathways that are commonly deregulated in cancer development. In this review, we will focus on the recent progress of research on miRNA-mediated regulation of apoptosis in breast cancer, discuss pro- and anti-apoptotic miRNAs (ApoptomiRs), miRNA targets, and their interaction with the p53 pathway. Finally, we will discuss current/future cancer prognostics and treatment targets based on these apoptotic regulators.

## Breast cancer and apoptosis

A critical balance between cell proliferation and apoptosis is essential for normal breast development. Accumulation of genetic or epigenetic mutations by various factors brings about disturbance in this balance leading to the development of breast tumors. Breast tumors are heterogeneous in their genetic composition and are histologically diverse based on receptor status, proliferation and differentiation markers that correlate to disease prognosis (Vakkala et al., 1999). The histological analyses guide clinicians in selecting adjuvant therapies for breast cancer treatment. Several studies have made attempts to correlate apoptosis and its associated molecular markers with known histologic and prognostic factors in breast cancer (Lipponen et al., 1994). A high apoptotic index has been correlated with increased tumor grade, aneuploidy, high mitotic index, negative status for estrogen receptor (ER), and progesterone receptor (PR), tumor necrosis and increased lymphocyte infiltration (Lipponen, 1999). The tumor suppressor gene, p53, and the BCL2 family have been widely studied in breast cancer. The mutant p53 correlates with negative ER status, high mitotic index, and the tumor grade, while BCL2 expression correlates to ER positive status, wild type p53, low mitotic index and low grade (Megha et al., 2002). Strikingly, expression of BCL2 is seen in ~80% of breast cancer patients. Contrary to expectations, high apoptotic index is significantly associated to poor prognosis while high BCL2 expression correlates to good prognosis of breast cancer patients (Silvestrini et al., 1996). Possible explanations may be that neither apoptotic index nor mutant p53 or BCL2 is independent predictor of survival in breast cancer patients. High apoptosis index also correlates to high proliferative index and negative ER status, which are poor prognostic factors. High apoptotic index may also put selective pressure on tumor cells leading to development of apoptosis resistant cells leading to tumor progression and decreased survival in patients (Frenzel et al., 2009). Similarly, BCL2, an estrogen regulated gene correlates with positive ER status, a good prognostic feature. Nevertheless, *in vitro* experiments with BCL2 overexpression or silencing in breast cancer cells confirm its pro-survival effects accounting for current pre-clinical and clinical trials involving BCL2 silencing for breast cancer treatment (Honma et al., 2015). Breast cancer cells show activation of the upstream apoptotic signaling such as an increase in Fas-L expression and presence of active caspases in cancer cells (Hengartner, 2001; Fulda and Debatin, 2006). However, upregulation of Inhibitors of apoptosis proteins (IAPs), X-linked inhibitor of apoptosis protein (XIAP), and Survivin inhibits activity of the caspases and blocks apoptotic signaling (Deveraux and Reed, 1999). Overall, the balance between the concentrations of pro- and anti-apoptotic proteins is an important regulatory factor for apoptotic regulation (Quail and Joyce, 2013).

## Pro- and anti-apoptotic miRNAs in breast cancer

Deregulation of apoptosis is an important step in cancer as it allows the genetically unstable cells to survive and accumulate further mutations that eventually lead to tumorigenesis (Elmore, 2007). One of the mechanism by which miRNAs influence tumor development is by regulation of proteins involved in the apoptotic process. miRNAs that function to promote or inhibit apoptotis are called pro- and anti-apoptotic miRNAs, respectively. Table 1 enlists various miRNAs that have been reported to function as pro- or anti-apoptotic miRNAs in breast cancer.

**Table 1:** **List of apoptosis-associated miRNAs in breast cancer**.

**miRNA**	**Target gene**	**Function of miRNA**	**Breast cancer cell lines/*in vivo* models**	**Citation**	**miRNA Cancer/normal**	
					**Fold Change**	***p*-value**
**PRO-APOPTOTIC miRs**						
miR-7-5p	PSME3	Inhibits cell proliferation and induces apoptosis *in vitro* and *in vivo*	MDA-MB-231, MCF7, and nude mice	Shi et al., 2015	2.08	S
miR-15/16	RPS6KB1	Inhibits cell proliferation and promotes cell cycle arrest and caspase-3 dependent apoptosis	MDA-MB-231, MCF7	Janaki Ramaiah et al., 2014	1.71, 1.44	S, S
miR-15a-5p	SNCG, CCNE1	Mediates cell cycle arrest and promotes cell apoptosis	MDA-MB-231, MDA-MB-231	Luo et al., 2013	1.71	S
miR-15a-3p	BCL2L1	Inhibits the expression of BCL2L1 and activates caspase-3/7 activity to promote apoptosis	MDA-MB-231	Druz et al., 2013	ND	ND
miR-16-5p	CCND1, BCL2, METTL13	Decreases cell growth and proliferation and induces apoptosis. Repression of METTL13 by miR-16 promotes apoptosis of cancer cells	MCF7	Rivas et al., 2012	1.44	S
mir-24-2-5p	BCL-2	mir-24-2-5p induces apoptosis by targeting BCL-2	MCF7	Srivastava et al., 2011	ND	ND
miR-26a	MTDH, EZH2, MCL1	Induces cell apoptosis and suppresses tumorigenesis *in vivo*	MCF7, MDA-MB-231, and nude mice	Zhang et al., 2011; Gao et al., 2013	0.72	S
		Repression of MCL1 by miR-26a suppresses cell growth and proliferation				
miR-26b	SLC7A11	Impairs viability and induces cell apoptosis	MCF7	Liu X. X. et al., 2011	0.83	S
miR-31	PRKCE (PKCϵ)	Enhances cell apoptosis, inhibits oncogenic NF-κB pathway and brings about indirect downregulation of BCL2	MCF10A, MDA-MB-231.	Körner et al., 2013	1.07	NS
miR-34a	BCL2, SIRT1, FRA1, LMTK3, AXL, NOTCH1, LDHA	Suppresses cells proliferation, glycolysis, migration, invasion, and induces apoptosis. Reduces cancer stem cell properties and increases sensitivity to doxorubicin treatment.	MCF7, MDA-MB-231, BT549, Hs578T, and nude mice	Mackiewicz et al., 2011; Li et al., 2013; Yang et al., 2013; Zhao et al., 2013; Park et al., 2014; Xiao et al., 2016	1.09	NS
miR-124	Ets-1	Induces cell apoptosis, reduces cell proliferation, and colony formation.	MCF7, MDA-MB-231.	Li W. et al., 2014	1.1	S
miR-125a	ELAVL1	Promotes apoptosis and inhibits cell growth and proliferation	MCF7	Guo et al., 2009	0.68	S
miR-125b	MUC1, EPO, EPOR, ENPEP, CK2-α, CCNJ, MEGF9, ERBB2, ARID3B	miR-125b promotes DNA damage-induced apoptosis and regulates cell motility	ZR-75-1, BT549, MCF7, MDA-MB-231	Rajabi et al., 2010; Akhavantabasi et al., 2012; Feliciano et al., 2013; Ferracin et al., 2013	0.27	S
miR-145	RTKN, c-Myc	Induces cell apoptosis and inhibits cancer cell growth	MCF7	Sachdeva et al., 2009; Wang et al., 2009	0.17	S
miR-146a/b	IRAK1, TRAF6	Mediates tumor suppression and triggers apoptosis	MCF7	Liu et al., 2015	1.11, 1.54	NS, S
miR-290-3p	ARID4B	miR-290 enhances ER signaling and increases apoptosis thereby suppressing breast cancer progression	6DT1, MVT-1	Goldberger et al., 2013	ND	ND
miR-486-5p	PIM-1	Suppresses cell proliferation *in vitro* and *in vivo*, induces G0/G1 arrest, and promotes apoptosis	MDA-MB-231, T47D and nude mice	Zhang et al., 2014	0.07	S
miR-497	Bcl-w (Bcl2L2)	Inhibits cellular growth and enhances apoptosis, promotes G0/G1 cell phase arrest	MCF7	Shen et al., 2012	0.38	S
miR-502-5p	TRAF2	Enhances early apoptosis and inhibits proliferation of breast cancer cells	MCF7, MDA-MB-231, and MCF-10A	Sun et al., 2014	ND	ND
miR-769-3p	NDRG1	Inhibits cell proliferation and enhances apoptosis	MCF7	Luo et al., 2014	ND	ND
miR-874	CDK9	Induces cell apoptosis and inhibits cell proliferation and brings about cell cycle arrest	MCF7, MDA-MB-231	Wang L. et al., 2014	0.77	S
miR-99a	mTOR	Induces cell apoptosis and suppresses cell viability	MCF7, MDA-MB-231	Hu et al., 2014	0.22	S
**ANTI-APOPTOTIC miRs**						
miR-21	BCL2, TIMP3, PDCD4, PTEN, TPM1, MASPIN, ANKRD46	miR-21 inhibition suppress both cell growth *in vitro* and tumor growth *in vivo* miR-21 promotes invasion in breast cancer cells	MCF7, MDA-MB-231, MDA-MB-435, and nude mice	Zhu et al., 2007; Frankel et al., 2008; Yang et al., 2009; Song et al., 2010; Wang et al., 2011; Yan et al., 2011	4.81	S
miR-24-3p	p27Kip1	Promotes cell proliferation and inhibits cell apoptosis	MDA-MB-435, MDA-MB-468	Lu et al., 2015	0.94	NS
miR-100	MTMR3	Antagonism of miR-100 led to G2/M cell-cycle arrest and induce apoptosis	SK-BR-3	He et al., 2015	0.23	S
miR-155	FOXO3a, SOCS1, RHOA	Induces cell survival and plays an important role in chemoresistance in breast cancer.	MCF7, MDA-MB-231	Kong et al., 2008, 2010; Jiang et al., 2010	2.24	S
miR-155	TP53INP1	miR-155 mediates cell proliferation and inhibits cell apoptosis	MCF7	Zhang et al., 2013	2.24	S
miR-96/182	PFN1, FOXO1	miR-182 promotes proliferation and invasion and inhibits apoptosis of breast cancer cells	MDA-MB-231, MCF7	Guttilla and White, 2009; Liu et al., 2013	miR-182 (5.31)	S
					miR-96 (7.35)	S
miR-196a	ANXA1	Enhances cell proliferation, colony formation and suppresses apoptosis	T47D, MDA-MB-453, MDA-MB-231	Luthra et al., 2008	5.12	S
miR-221/222	PTEN, PUMA, CASP3, p27Kip1	Enforced expression of miR-221/222 promotes breast cancer cell proliferation, migration and invasion and inhibits apoptosis by targeting and blocking caspase-3 expression	MCF7, SKBR3, HCC1500, MDA-MB- 231,	Miller et al., 2008; Zhang et al., 2010; Ergun and Oztuzcu, 2014; Li et al., 2016	miR-221 (0.81), miR-222 (0.79)	S, S
miR-210	RAD52, GPD1L	miR-210 overexpression inhibits apoptosis	MCF7	Crosby et al., 2009; Fasanaro et al., 2009	5.46	S
miR-504	P53	miR-504 overexpression reduces p53 mediated apoptosis and cell cycle arrest in response to stress	MCF7 and nude mice	Hu et al., 2010	0.73	S

### Pro-apoptomiRs

A total of 22 miRNAs have been reported so far to be involved in the induction of apoptosis suggesting them to be tumor suppressors. Tumor suppressor miRNAs prevent tumor progression by negatively regulating the expression of genes that promote cell proliferation, differentiation, migration, or apoptosis. We evaluated the expression of pro-apoptomiRs in breast cancer patients using StarBase software that analyzes TCGA data (Li J. H. et al., 2014). The data set included 683 breast cancer patients and 87 normal samples. Interestingly, we found that miR-26a, -26b, -125a, -125b, -145, -486-5p, -497, -874, and -99a that are known to function as proapoptomiRs were significantly downregulated in breast cancer patients. In the following section, we have discussed some pro-apoptotic miRNAs.

#### miR-15/16 cluster

miR-15/16 was shown to inhibit cell proliferation and promote cell cycle arrest and apoptosis in various breast cancer cell lines (Rivas et al., 2012; Druz et al., 2013; Luo et al., 2013; Janaki Ramaiah et al., 2014). The members of miR-15/16 cluster negatively regulate the anti-apoptotic protein BCL2, cell cycle regulators CCND1, CCNE1 and other proteins RPS6KB1, SNCG and METTL13 in breast cancer (refer Table 1).

#### miR-26a/b

miR-26a is downregulated in breast cancer specimens and cell lines and its transient transfection initiates apoptosis of breast cancer cell line, MCF7. Oncogenes, MTDH, and EZH2 (a chromatin regulator), were identified as direct targets of miR-26a. MCF7 xenografts with exogenous miR-26a show that a decrease in expression of both MTDH and EZH2 is accompanied by an increase in apoptosis (Zhang et al., 2011). MCL1, an anti-apoptotic member of the BCL2 family and SLC7A11, an amino acid transporter are other targets of miR-26a (Gao et al., 2013). Silencing of SLC7A11 mimics miR-26b aroused viability impairment and apoptosis in MCF7 cells.

#### miR-34a

Several groups have independently reported pro-apoptotic role of miR-34a in breast cancer (Mackiewicz et al., 2011; Li et al., 2013; Yang et al., 2013; Zhao et al., 2013; Park et al., 2014; Xiao et al., 2016). miR-34 overexpression suppresses cell proliferation, glycolysis, metastasis, invasion, stem cell properties, and promotes apoptosis and sensitivity to doxorubicin treatment in breast cancer (Mackiewicz et al., 2011; Li et al., 2013; Yang et al., 2013; Zhao et al., 2013; Park et al., 2014; Xiao et al., 2016). miR-34a is transcriptionally regulated by p53 and targets SIRT1 (a modulator of p53 activity) thus making a mir-34-p53 positive feedback loop (Li et al., 2013). Other targets of miR-34a are BCL2, FRA1, LMTK3, AXL, NOTCH1 and LDHA. miR-34a/AXL interaction reduces phospho-AKT expression, and impairs the motility of triple-negative breast cancer cells (Mackiewicz et al., 2011). miR-34a suppresses MCF7 cell proliferation *in vitro* and *in vivo* by targeting LMTK3 in an estrogen dependent manner (Zhao et al., 2013). Overall, miR-34a functions as a tumor suppressor in breast cancer as also reported for other cancers.

#### miR-125a/b

miR-125a/b promotes apoptosis and inhibits cell growth and proliferation. miR-125a functions as a tumor suppressor for breast cancer, with HuR as a direct and functional target (Guo et al., 2009). miR-125b perform its tumor suppressor function via the direct targeting of the 3′-UTRs of *ENPEP, CK2*-α, *CCNJ, MUC1*, and *MEGF9* mRNAs. miR-125b regulates cell motility by targeting ERBB2 and ARID3B (Rajabi et al., 2010; Akhavantabasi et al., 2012; Feliciano et al., 2013; Ferracin et al., 2013). Overexpression of miR-125b markedly inhibits Taxol-induced cell cytotoxicity in MDA-MB-435, MDA-MB-231, and BT474 breast cancer cells by targeting 3′UTR of *BAK1* mRNA, which is an effector of taxol induced apoptosis (Zhou et al., 2010).

## Anti-apoptomiRs

A total of 11 miRNAs have been shown to inhibit apoptosis suggesting them to be oncomiRs in breast cancer. Among these miR-21, -155, -96, -182, -196a, and -210 were found to be highly upregulated in breast cancer patients (Table 1).

### miR-21

miRNA 21 is overexpressed in many cancers and has been shown to have oncogenic activity. Si et al., 2007 showed that miR-21 knock down inhibits MCF7 cell growth *in vitro* and suppresses MCF7 cell derived breast tumor growth in a murine xenograft model (Si et al., 2007). This was associated with increased apoptosis and decreased cell proliferation by downregulation of anti-apoptotic BCL2. miR-21 was also shown to promote breast cancer cell invasion. Overall, miR-21 functions as an oncomiR and modulates tumorigenesis through regulation of genes such as ANKRD46, BCL2, TIMP3, PDCD4, PTEN, TPM1, and MASPIN in breast cancer (Zhu et al., 2007; Frankel et al., 2008; Yang et al., 2009; Song et al., 2010; Wang et al., 2011; Yan et al., 2011).

### miR-155

miR-155 exerts its anti-apoptotic activity by downregulating FOXO3a for cell survival (Kong et al., 2010). Active form of FOXO3a resides in the nucleus and induces cell death by up-regulation of apoptotic proteins, such as BIM, p27, BNIP3, and 24p3 and repression of anti-apoptotic proteins, FLIP, and BCL-xL (Kong et al., 2010). miR-155 also targets TP531NP1, SOCS1, and RHOA and inhibits apoptosis and chemosensitivity in MCF7 cells (Kong et al., 2008, 2010; Jiang et al., 2010; Zhang et al., 2013).

### miR-210

miR-210 is a hypoxia inducible miRNA that functions as an oncomiR in breast cancer. High miR-210 expression is a poor prognostic factor in breast cancer patients. miR-210 inhibition promotes apoptosis in breast cancer. It is known to target DNA repair protein RAD52 and a protein involved in mitochondrial function regulation, GPD1L (Crosby et al., 2009; Fasanaro et al., 2009).

### miR-221/222

miR-221/222 are frequently up-regulated in human epithelial cancers. Enforced expression of miR-221/222 promotes breast cancer cell proliferation, migration, and invasion via targeting PTEN/AKT pathway. In breast cancer miR-221/222 directly targets pro-apoptotic proteins PUMA and CASP3 and cell cycle inhibitor, p27Kip1 to show its oncogenic effect (Zhang et al., 2010). miR-221/222 inhibition results in an increase in mitochondrial membrane potential and caspase-3/7 activity thereby leading to apoptosis in the A549 and MCF7 cell lines (Zhang et al., 2010).

## Targets of apoptomiRs

**Figure 1:**
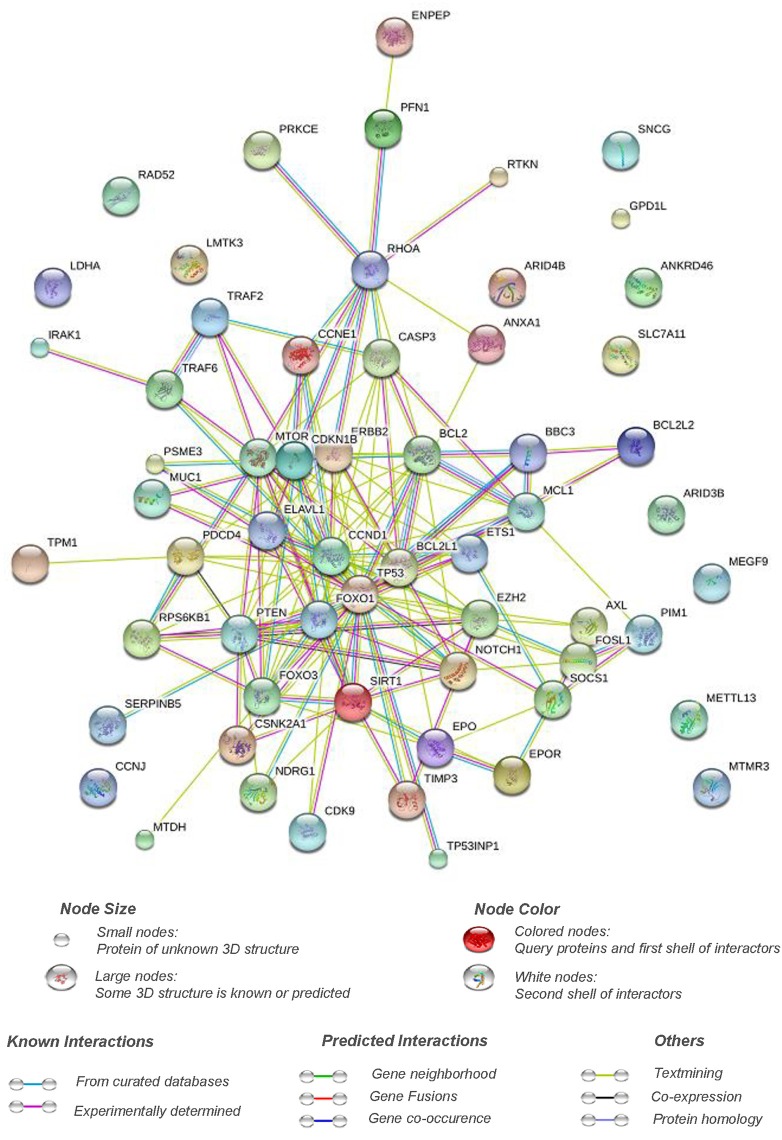
**Pathway interaction network of targets of apoptomiRs in breast cancer**. STRING interaction database (Von Mering et al., 2005) was used at setting of high confidence (0.7) to generate the interaction network of the list of the targets of apoptomiRs in breast cancer (Zhu et al., 2007; Frankel et al., 2008; Kong et al., 2008; Luthra et al., 2008; Miller et al., 2008; Crosby et al., 2009; Fasanaro et al., 2009; Guo et al., 2009; Guttilla and White, 2009; Sachdeva et al., 2009; Wang et al., 2009; Yang et al., 2009; Hu et al., 2010; Jiang et al., 2010; Kong et al., 2010; Rajabi et al., 2010; Song et al., 2010; Zhang et al., 2010; Liu X. X. et al., 2011; Mackiewicz et al., 2011; Srivastava et al., 2011; Wang et al., 2011; Yan et al., 2011; Zhang et al., 2011; Akhavantabasi et al., 2012; Rivas et al., 2012; Shen et al., 2012; Druz et al., 2013; Feliciano et al., 2013; Ferracin et al., 2013; Gao et al., 2013; Goldberger et al., 2013; Körner et al., 2013; Li et al., 2013; Liu et al., 2013; Luo et al., 2013; Yang et al., 2013; Zhang et al., 2013; Zhao et al., 2013; Ergun and Oztuzcu, 2014; Hu et al., 2014; Janaki Ramaiah et al., 2014; Luo et al., 2014; Li W. et al., 2014; Park et al., 2014; Sun et al., 2014; Wang L. et al., 2014; Zhang et al., 2014; He et al., 2015; Liu et al., 2015; Lu et al., 2015; Shi et al., 2015; Li et al., 2016; Xiao et al., 2016). The implication of the various connective lines based on different data sources are shown below the figure.

A total of 61 genes have been validated as direct targets of apoptomiRs. Interestingly, ~36% of these targets were found to be altered at the transcript (BCL2, CCNE1, CSKN2A1, ELAVL1, ERBB2, ETS1, EZH2, FOXO1, IRAK1, MUC1, MYC, NDRG1, PDCD4, SERPINB5, RPS6B1, SNCG, TIMP3, TP53, and TP53INP1) or the protein (BCL2, CASP3, CCNE1, CDKN1B, ERBB2, MUC1, MYC, PTEN, SERPINB5, TP53) levels in breast cancer based on analyses using genes to system breast cancer (G2SBC) database (Mosca et al., 2010). Whether the effects of the miRNAs on apoptosis are mediated through these targets has not been fully elucidated. Pathway analyses of the target genes of apoptomiRs using Reactome pathway browser identified transcriptional regulation by TP53 (CCNE1, CDKN1B, mTOR, CDK9, CSKN2A1, NDRG1, PTEN, PUMA, TP53, TP53INP1); signaling by nerve growth factor, NGF (IRAK1, ERBB2, CASP3, CDKN1B, RHOA, mTOR, FOXO1, PSME3, FOXO3, PRKCE, TRAF6, PTEN); and PI3K/AKT activation (CDKN1B, ERBB2, RHOA, mTOR, FOXO1, FOXO3, PTEN) as the top three overrepresented pathways (Joshi-Tope et al., 2005). NGF is known to be overexpressed in ~80% of breast tumor biopsies and has been implicated in enhanced survival, proliferation, migration, and inhibition of apoptosis in breast cancer (Molloy et al., 2011). Similarly, the pro-proliferative and anti-apoptotic effects of PI3K/AKT pathway are well documented. A protein interaction network analyses using STRING database (Von Mering et al., 2005) reveals that a strong interaction network with a high confidence (0.7) exists between the validated targets of the apoptomiRs suggesting that this set of the genes are biologically related (Figure 1).

Interestingly, around 40% of the apoptomiR targets (BCL2, BCL2L1, BCl2L2, FOSL1, NOTCH1, TIMP3, TRAF2, TRAF6, ANXA1, CASP3, CDKN1B, FOXO1, FOXO3, IRAK1, MCL1, PTEN, PIM1, PSME3, PRKCE, PUMA, RHOA, SIRT1, TP53, TP53INP1, ERBB2, and ETS1) were found to be associated with regulation of apoptosis based on functional annotation clustering using DAVID microarray software (Huang et al., 2009a,b). Several of these belong to the p53 network or BCL2 family of proteins involved in control of apoptosis.

**Figure 2:**
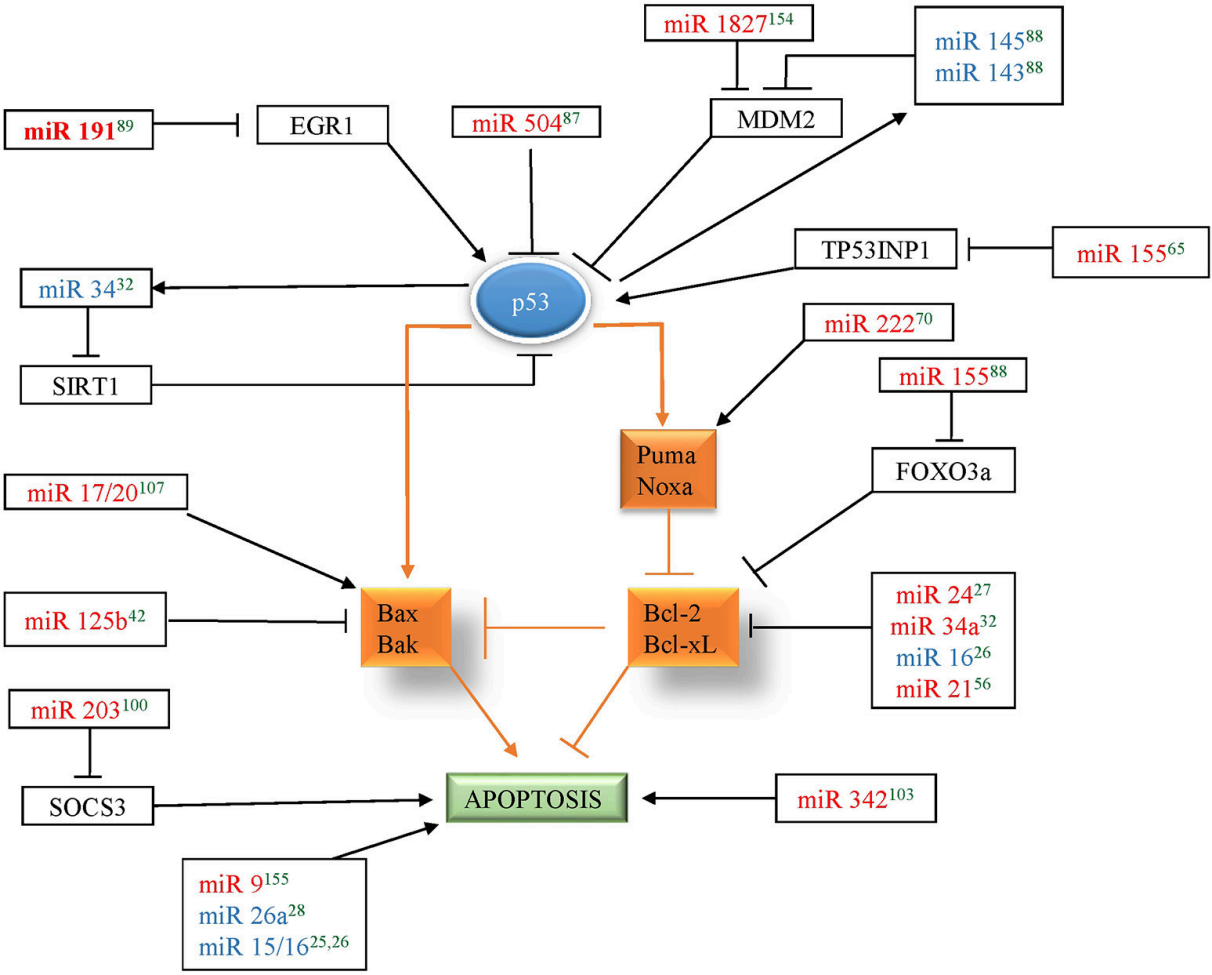
**miRNAs in red color correspond to the miRNAs that regulate p53 in breast cancer and miRNAs in blue color corresponds to the miRNAs that are regulated by p53 in breast cancer**. Arrow indicates activation and bar indicates inhibition. The references for each miRNA are marked as superscript in green color. ^25^(Druz et al., 2013), ^26^(Rivas et al., 2012), ^27^(Srivastava et al., 2011), ^28^(Zhang et al., 2011), ^32^(Li et al., 2013), ^42^(Feliciano et al., 2013), ^56^(Frankel et al., 2008), ^65^(Zhang et al., 2013), ^70^(Zhang et al., 2010), ^87^(Hu et al., 2010), ^88^(Okamura et al., 2001), ^89^(Nagpal et al., 2013), ^100^(Ru et al., 2011), ^103^(He et al., 2013), ^107^(Yu et al., 2014), ^154^(Zhang et al., 2016), ^155^(Zhou et al., 2015).

p53, is a tumor suppressor protein that mediates cellular responses to various stress signals like DNA damage, hypoxia, or aberrant oncogene expression by promoting cell-cycle checkpoints, DNA repair, cellular senescence, and apoptosis. In breast cancer, TP53 mutations have been associated with poor response to various therapies and thus poor prognosis. Several miRNAs were found to be associated with the p53 network by targeting p53 directly or indirectly (by targeting its upstream regulators or downstream mediators) to influence apoptosis in breast cancer. miR-504 was shown to directly target p53 and thus, inhibit p53 dependent apoptosis in breast cancer (Hu et al., 2010). MDM2 is an E3 ubiquitin-protein ligase that mediates ubiquitination of p53, leading to its proteasomal degradation and nuclear export. miR-143/145, and miR-1827 were found to directly target MDM2 in breast cancer and thus enhance p53-mediated stress responses, including apoptosis and senescence (Okamura et al., 2001). TP53INP1, SIRT1, and EGR1, regulators of p53 transcriptional activity, have also been shown to be direct targets of apoptomiRs- miR-155, miR-34, and miR-191, respectively, in breast cancer (Li et al., 2013; Nagpal et al., 2013; Zhang et al., 2013). Similarly, several miRNAs (miR-9, miR-17/20, 21, 125b, 155, 203, 222, and 342) affect the levels of p53 downstream genes to establish their effect on apoptosis. Additionally, p53 has been shown to induce the levels of several miRNAs involved in regulation of apoptosis in breast cancer. A total of six tumor suppressor miRNAs (miR-143, miR-145, miR-15, miR-16, miR-26a, and miR-34) have been shown to be upregulated by p53 in breast cancer (Sachdeva et al., 2009; Gao et al., 2013; Li et al., 2013; Janaki Ramaiah et al., 2014). Since miR-143/145 regulate MDM2 (regulator and transcriptional target of p53) and are also regulated by p53, it indicates existence of miR-143/145-MDM2-p53 feedback loop in breast cancer that controls cell proliferation and apoptosis (Okamura et al., 2001). Similarly, miR-34 is induced by p53 and targets SIRT1 (a modulator of p53 activity) thus making a mir-34-p53 positive feedback loop (Yamakuchi et al., 2008). Overall, apoptomiRs emerge as important regulators of the p53 network at multiple levels to regulate apoptosis in breast cancer (Figure 2).

A number of BCL2 family members were found to be targeted by apoptomiRs by various groups. BCL2 family consists of both pro and anti-apoptotic proteins. BCL2, an antiapototic protein, was found to be targeted by four miRNAs (miR-21, -24, -34a, and -16) indicating that its levels are kept under tight control in breast tissue. Overexpression of miR-24, 34a, and 16 reduces BCL2 expression that leads to apoptosis through disruption of the mitochondrial membrane potential and the release of cytochrome C (Srivastava et al., 2011; Rivas et al., 2012; Li et al., 2013). Other anti-apoptotic members of the BCL2 family are also directly targeted by apoptomiRs- (BCL2L1- mir-15a-3p, BCL2L2- miR-497, MCL1- miR-26a; Shen et al., 2012; Druz et al., 2013; Gao et al., 2013). The level of BAK1, a pro-apoptotic BCL2 family protein, was found to be a direct target of oncomiR, miR-125b. PUMA, belonging to the BH3-only subgroup of BCL2 family protein was also found to be targeted by miR-221/222 (Zhang et al., 2010). miR-17/20 transduction of MCF7 cells has been shown to induce p53 and apoptosis characterized by activation of BAX, release of cytochrome C, and induction of BCL-X(S) in response to DNA damaging agents, like doxorubicin and tamoxifen. Overall, a multipronged attack by miRNAs seems to directly or indirectly target BCL2 family proteins or downstream targets in breast cancer.

CASP3 is a mediator of apoptosis that gets activated in apoptotic cell by both death ligand (extrinsic) and mitochondrial (intrinsic) pathways. It was found to be a direct target of miR-221/222 in breast cancer (Miller et al., 2008). Several cell cycle related genes (CCND1, CCNE1, CCNJ, CDK9, CDKN1B, and PSME3) also feature in the list suggesting important roles these apoptomiRs may play in maintaining balance between apoptosis and cell cycle. Finally, apoptomiRs target crucial genes involved mainly in the apoptotic or cell cycle related pathways in breast cancer. Fishing out more targets using advanced technologies will likely widen the range of the pathways affected by these targets.

### miRNAs and drug resistance in breast cancer

The commonly used drugs for breast cancer treatment are Cyclophosphamide, Docetaxel, Doxorubicin, Epirubicin, Methotrexate, Paclitaxel, Cisplatin, Carboplatin, 5 Fluorouracil, Gemcitabine, etc. The patients are also given treatment based on the receptor status. The patients are tested for ER, Her2, and PR status. ER+ve patients receive tamoxifen (antagonist of ER signaling) and Her2+ve patients receive trastuzumab (herceptin), a monoclonal antibody against Her2, included in the chemotherapy regimen (Osborne, 1998; Ariazi et al., 2006). While the response of breast cancer patients to radiation, drug, or hormonal treatment has considerably improved the patient disease free survival and overall survival, chances of recurrence still remain. Since apoptosis mediated cancer cell death remains central to all the treatment strategies, the recurrence has been attributed to the development of resistance to apoptotic death and maintenance of cell viability by undergoing viable cellular responses such as cellular senescence (generates secretomes which can directly enhance the malignant phenotype) or autophagy (a state of reversible dormancy making it possible for cancer cells to survive and regrow at a later stage; Degenhardt et al., 2006).

Several mechanisms of chemoresistance in breast cancer have been reported in breast cancer such as upregulation of the ATP-binding cassette (ABC) transporters, drug inactivation or detoxification, alterations in genes related to cell cycle, apoptosis or DNA repair enzymes, epigenetic modifications, and activation of signaling pathways related to the progression of cancer (Baker et al., 2005; Pogribny et al., 2010). Recently, alterations of miRNA levels too have been linked to the development of drug resistance in breast cancer (Kovalchuk et al., 2008; Baylin, 2011). Very few miRNAs (miR-17/20, -21, -34a, -125b, -181a, -203, -218, -221, -222, -342) have been reported till date to affect the sensitivity to drugs (paclitaxel, cisplatin, doxorubicin, and taxol) or hormonal (tamoxifen and trastuzumab) treatment in various breast cancer cell lines (Table 2). These miRNAs affect the drug sensitivity by interfering in apoptotic death by affecting the levels of various apoptotic regulators (TP53, BCL2, BAX, BAK1, SURVIVIN, NOTCH1), members or regulators of AKT signaling pathway (AKT1, PTEN), ID4, NOTCH1 and SOCS3. Considering that the levels of several miRNAs change in response to drug treatment or are differentially regulated between sensitive versus resistant breast cancer cell lines, it is very likely that the list of miRNAs associated with drug resistance will expand. Some of these miRNAs may also function as biomarkers in predicting and monitoring drug response in breast cancer patients (Nana-Sinkam and Croce, 2013). These miRNAs are also being evaluated for their use in combination therapies to counteract drug resistance (Nana-Sinkam and Croce, 2013).

**Table 2:** **miRNAs associated with drug resistance in breast cancer**.

**miRNA**	**Drug**	**Targets**	**Cell lines/*in vivo* models**	**Citation**
miR-125b	Paclitaxel (Taxol)	Bcl-2 antagonist killer 1 (Bak1) is a direct target of miR-125b. miR-125b confers Taxol resistance through suppression of Bak1 expression	MDA-MB-435, MDA-MB-231, MDA-MB-436, MCF7, SKBR3, and BT474	Zhou et al., 2010
miR-203	Cisplatin	Knockdown of miR-203 sensitized human breast cancer MCF7 cells to cisplatin-mediated apoptotic cell death	MCF7, ZR-75, and MDA-MB-231	Ru et al., 2011
		SOCS3 is a novel target of miR-203 and plays an important role in cisplatin sensitivity of breast cancer cells		
miR-21	Doxorubicin(ADR)	Dysregulation of miR-21 plays critical roles in the ADR (doxorubicin) resistance of breast cancer via targeting PTEN	MCF7	Wang et al., 2011
MIR-34a	Doxorubicin(ADR)	Ectopic miR-34a expression reduces cancer stem cell properties and increases sensitivity to doxorubicin treatment by directly targeting NOTCH1	MCF7	Li et al., 2012
miR-342	Tamoxifen	Overexpression of miR-342 upregulates the expression of ER-α mRNA and sensitizes the MCF7 cells to tamoxifen. miR-342 is down-regulated in breast tumors resistant to Tamoxifen. Reintroduction of miR-342 sensitizes refractory breast tumor cells to tamoxifen therapy. ID4 was identified as a putative target of miR-342	MCF7, BT20, MDA-MB-231,T47D, HCC1937	He et al., 2013; Crippa et al., 2014
miR-221/222	Trastuzumab	miR-221 mediated downregulation of PTEN confers trastuzumab resistance of HER2-positive breast cancers	SKBR3 and nude mice	Ye et al., 2014
miR-181a	Adriamycin	miR-181a regulates the chemosensitivity to Adriamycin by targeting BCL2 in MCF7 and MCF7/ADR cells	MCF7	Zhu et al., 2013
miR-17/20	Doxorubicin and Tamoxifen	miR-17/20 sensitized breast cancer cells to chemotherapy-induced apoptosis requires AKT1	MCF7	Yu et al., 2014
miR-218	Doxorubicin and Taxol	miR-218 is involved in the development of multi drug resistance in breast cancer cells via targeting SURVIVIN leading to evasion of apoptosis	MCF7 and nude mice	Hu et al., 2015

## ApoptomiRs as biomarkers and therapeutic targets in breast cancer

**Figure 3:**
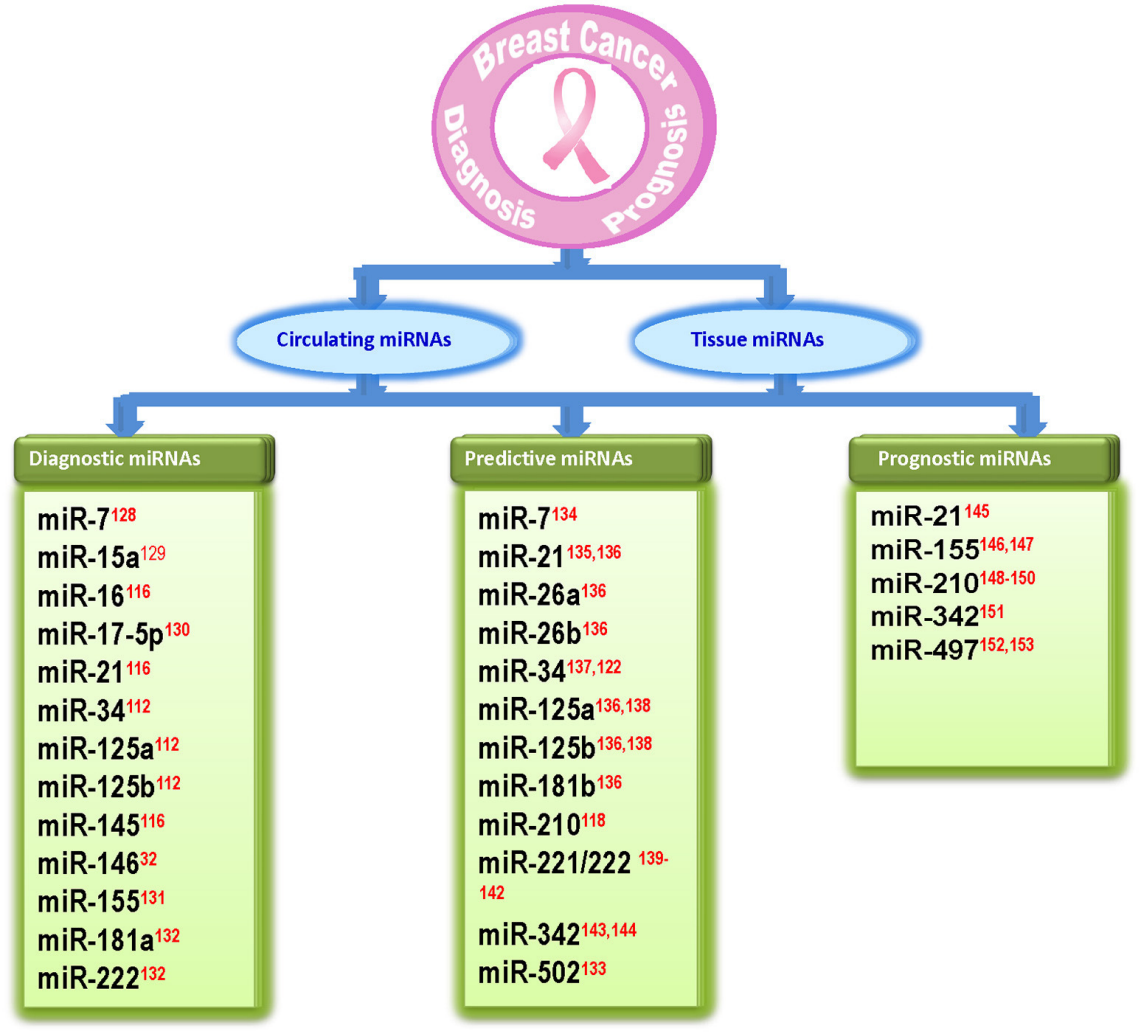
**Diagram showing apoptosis related miRNAs that are identified as biomarkers in breast cancer**. The references for each miRNA are marked as superscript in red color. ^32^(Li et al., 2013), ^112^(Iorio et al., 2005), ^116^(Ng et al., 2013), ^118^(Jung et al., 2012), ^122^(Kato et al., 2009), ^128^(Foekens et al., 2008), ^129^(Kodahl et al., 2014), ^130^(Volinia et al., 2006), ^131^(Wang F. et al., 2014), ^132^(Godfrey et al., 2013), ^133^(Lyng et al., 2012), ^134^(Chen and Bourguignon, 2014), ^135^(Mei et al., 2010), ^136^(Maillot et al., 2009), ^137^(Stankevicins et al., 2013), ^138^(Valabrega et al., 2007), ^139^(Zhao et al., 2008), ^140^(Wei et al., 2014), ^141^(Gan et al., 2014), ^142^(Rao et al., 2011), ^143^(He et al., 2013), ^144^(Cittelly et al., 2010), ^145^(Lee et al., 2011), ^146^(Song et al., 2012), ^147^(Kong et al., 2014), ^148^(Volinia et al., 2012), ^149^ (Li M. et al., 2014), ^150^(Wang J. et al., 2014), ^151^(Leivonen et al., 2014), ^152^(Shen et al., 2012), ^153^(Wang et al., 2013).

Early detection of breast cancer is important to reduce the risk of disease. Current diagnostic methods for early detection of breast cancer are still limited to some procedures such as tissue biopsies and histological examination using mRNA or protein biomarkers. However, there is still need for sensitive and specific markers. As miRNAs have small size, high specificity, and greater stability, they can be used as biomarkers with diagnostic (based on their ability to discriminate between normal and breast cancer patients), predictive [which are involved in response (sensitivity/resistance) to conventional breast cancer therapeutic strategies] or prognostic (which predict patient overall or disease-free survival) potential (Krutovskikh and Herceg, 2010; Abba et al., 2012; Schooneveld et al., 2015). Several studies have shown the role of miRNAs in diagnosis and prognosis of breast cancer. Iorio et al. (2005) identified a 13 miRNA signature that could differentiate breast cancer from normal breast tissue with 100% accuracy (Iorio et al., 2005). Blenkiron et al. (2007) identified 133 miRNAs that displayed aberrant expression levels in breast tumor tissues compared with normal breast tissues (Blenkiron et al., 2007). Recently several reports suggested that circulating miRNAs are highly stable in serum/plasma and levels of some miRNAs are specifically elevated in patients with breast cancer (Heneghan et al., 2010). In a recent study, nine circulating miRNAs were shown to be capable of discriminating between early-stage breast cancer and healthy controls (Kodahl et al., 2014). Another study reported three significantly overexpressed serum miRNAs in breast cancer patients as compared to breast cancer free controls (Ng et al., 2013). Recently, Bertoli et al. (2015) and Schooneveld et al. (2015) reported circulating and non-circulating miRNAs as breast cancer biomarkers. Several of the apoptomiRs have also been shown to serve as biomarkers in breast cancer (Abba et al., 2012; Bertoli et al., 2015; Figure 3). While some apoptomiRs have only diagnostic (miR-15a/16, -17, -181a, -145, -146) or predictive (miR-26a/b, -181b, -502) or prognostic (miR-497) potential, some have been proposed to serve as biomarkers for any two (miR-7, -34, -125a/b, -155, -222, -342) or all the three purposes (miR-21). Based on their presence and high stability in patient serum, capacity to reflect tumor status, and high clinical outcome, some apoptomiRs (miR-155, -21, -16, -222, and -210) are present as circulating miRNAs and can be used as potential biomarkers for breast cancer. miR-210 is a hypoxia regulated anti-apoptotic miRNA in breast cancer and high miR-210 baseline expression has been associated with poor prognosis and resistance to trastuzumab-included chemotherapy (Jung et al., 2012) High miR-155 levels too have been reported in tumor tissues sections and the serum of breast cancer patients and have been correlated to poor prognosis (Kong et al., 2014). Similarly, miR-21 upregulation is associated with taxol resistance and poor prognosis in breast cancer cells (Mei et al., 2010). miR-34 has been proposed to serve as a predictive marker for response to radiotherapy (Stankevicins et al., 2013). Kato et al. reported that low levels of miR-34a rendered breast cancer cells more resistant to radiotherapy (Kato et al., 2009).

While successful miRNA delivery still remains a challenge, the prospects of using miRNAs as means and targets for breast cancer therapy remain attractive. There are two lines of action for therapeutic use of miRNAs: miRNA antagonists for miRNA inhibition and miRNA mimics for miRNA replacement therapy (Bader, 2012; Thorsen et al., 2012). Based on *in vitro* studies several miRNAs have reached preclinical trials and two miRNAs, have entered clinical trials. A locked nucleic acid-based antisense oligonucleotide against miR-122 (Miravirsen), successfully completed phase I clinical study and is undergoing phase II clinical trials for treatment of chronic hepatitis C virus infection (Janssen et al., 2013). In preclinical studies, Liu et al. reported that the injection of miR-34a mimic extended the survival of tumor-bearing mice (Liu C. et al., 2011). Another study demonstrated that systemic administration of miR-34 in a pancreatic xenograft cancer model significantly inhibited tumor growth and induced cancer cell apoptosis (Hu et al., 2013). A clinical trial using miR-34 mimic (MRX34) has already entered phase I clinical trial in patients with unresectable primary liver cancer or metastatic cancer with liver involvement (ClinicalTrials.gov Identifier: NCT01829971). Considering that miR-34 plays a major role in breast tumorigenesis and shows promising results in preclinical trials, miR-34 may soon enter clinical trials for treatment of breast cancer patients.

## Conclusions

Mechanisms of cell death and its regulation during initiation, progression, and treatment of breast cancer are complex and still remain partially understood. The role of miRNAs as critical mediators of apoptotic cell death has been a revelation in the past decade and has given us means to manipulate cell death for breast cancer treatment or for disease monitoring. The current impediments for successful treatment are miRNA delivery issues, concerns over off-target effects and our incomplete knowledge of the functions of these miRNAs. Many of these miRNAs may have uninvestigated roles in other forms of cell death that may change the output based on the balance between the desired (apoptosis) and undesired (autophagy and necrosis) forms of cell death for breast cancer treatment. Nevertheless, miRNA delivery issues are slowly getting resolved, miRNAs are also being considered as means to target multiple aberrant pathways and certainly preclinical and clinical trials of some of these miRNAs look promising. The pool of apoptomiRs in breast cancer is likely to get bigger with advanced technologies such as large scale miRNA functional screens using 3D tumor models, thus giving us more probable candidates as novel targets for breast cancer therapy.

## Author contributions

RK conceptualized the theme of the review and has written the review. SS compiled all the data on pro- and anti-apoptotic miRNAs and their correlation to drug resistance and has contributed in writing the review. PP and SA compiled the data of miRNA and p53 regulatory loop and contributed toward writing this section.

### Conflict of interest statement

The authors declare that the research was conducted in the absence of any commercial or financial relationships that could be construed as a potential conflict of interest. The reviewer KT and handling editor declared their shared affiliation, and the handling editor states that the process nevertheless met the standards of a fair and objective review.
